# Impurity Tolerance in LiFePO_4_ Cathodes: Contrasting Structural, Electronic, and Electrochemical Roles of Residual Ni and Cr

**DOI:** 10.1002/advs.75774

**Published:** 2026-05-22

**Authors:** Minjin Kim, Hyerin Jeon, Jinhee Lee, Jinsub Choi

**Affiliations:** ^1^ Department of Chemistry and Chemical Engineering Inha University Incheon Republic of Korea; ^2^ Department of Secondary Battery Convergence Engineering Inha University Incheon Republic of Korea

**Keywords:** chromium impurities, impurity engineering, LiFePO_4_ cathode, nickel substitution, recycled precursors

## Abstract

The synthesis of high‐performance cathode materials has traditionally relied on high‐purity precursors, posing challenges for large‐scale battery recycling, where complete removal of transition‐metal impurities is often impractical. This issue is particularly relevant for LiFePO_4_ (LFP) cathodes, as recycled Fe sources inevitably contain residual elements such as Ni and Cr. Here, the structural and electrochemical effects of residual Ni and Cr impurities in LFP are systematically investigated using combined experimental characterization and density functional theory (DFT) calculations. The results show that impurity tolerance in LFP strongly depends on elemental identity and concentration. Low levels of Ni (∼1 mol%) can be accommodated within the lattice with minimal structural disruption, leading to improved charge‐transfer kinetics and rate performance. In contrast, higher Ni contents induce lattice distortion and increased Li–Fe antisite defects, with the transition from beneficial to detrimental behavior occurring between 1 and 2 mol% Ni. Cr impurities exhibit fundamentally different behavior. Their incorporation is thermodynamically unfavorable, resulting in inhomogeneous distribution and segregation of electrochemically inactive Cr_2_O_3_ phases. These phases introduce resistive and diffusion‐blocking heterogeneities, leading to increased polarization and degraded electrochemical performance. Overall, this study establishes an impurity‐tolerance framework that provides practical guidelines for sustainable LFP synthesis from recycled resources.

## Graphical abstract

Impurity tolerance in LiFePO_4_ cathodes varies strongly with impurity type and content. Low Ni levels are accommodated within the lattice with minimal structural disruption, whereas higher Ni contents introduce antisite disorder. In contrast, Cr impurities segregate from the LiFePO_4_ framework, blocking lithium‐ion pathways and leading to inferior electrochemical performance.

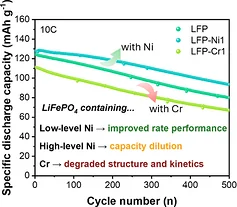

## 1 Introduction

The demand for electric vehicles and large‐scale energy storage systems is surging worldwide, driven by the rapid expansion of renewable energy infrastructure [1, 2]. This rapid growth in energy demand has intensified concerns regarding the long‐term stability of raw‐material supply chains in the battery industry. Critical metals such as lithium, nickel, and cobalt are subject to volatile pricing and geographical concentration, motivating a transition toward more resilient and sustainable resource‐management strategies [3, 4]. In response, the upcycling of industrial byproducts and end‐of‐life battery materials has emerged as a key pathway toward a circular battery economy. For example, silicon waste from semiconductor manufacturing has been repurposed for high‐capacity anodes [5, 6], while spent Ni‐rich layered cathodes (e.g., NCM) are increasingly recycled via hydrometallurgical processes to recover transition metals [7, 8, 9].

At the same time, the growing reliance on recycled resources challenges a long‐standing assumption in cathode‐material synthesis—namely, that high electrochemical performance necessarily requires the use of high‐purity precursors. In practical recycling processes, impurity elements are difficult to eliminate completely without resorting to energy‐intensive and costly purification steps [7, 10], raising fundamental questions regarding impurity tolerance in battery materials. In this context, recent studies have demonstrated that battery materials derived from industrial waste or recycled precursors can exhibit competitive electrochemical performance even in the presence of residual impurities, highlighting the feasibility of impurity‐inclusive material design [11].

Among various industrial waste streams, stainless‐steel (STS) scrap represents a promising yet underutilized resource. With over 50 million tons generated annually, most STS scrap is currently downcycled through remelting. However, its high iron content (>70 wt.%), together with significant fractions of Ni and Cr, suggests substantial potential for chemical valorization [12, 13, 14]. Recently, integrated recovery routes combining pyrometallurgical treatment and electrochemical leaching have been proposed to extract iron sulfate (FeSO_4_) and nickel sulfate (NiSO_4_) from STS scrap. While FeSO_4_ serves as a key precursor for LiFePO_4_ (LFP) synthesis, STS‐derived Fe sources often contain residual Ni and Cr impurities [10, 15, 16]. Conventionally, complete removal of these elements is pursued through rigorous purification, despite the associated increases in energy consumption, cost, and environmental burden. This practice raises a critical question: is exhaustive purification always necessary, or can certain impurities be tolerated—or even utilized—within the LFP lattice?

Importantly, STS scrap is not the only realistic source of impurity‐containing Fe precursors. In lithium‐ion battery recycling, black mass is typically produced via mechanical shredding processes that do not discriminate between different cathode chemistries. As a result, LiFePO_4_ can be physically mixed with Ni‐rich layered oxides such as NCM or NCA within the same recycled stream [17, 18]. During subsequent hydrometallurgical processing, Ni originating from Ni‐based ternary cathodes can be co‐dissolved together with Fe‐containing species, complicating the purification of Fe‐based precursor solutions and often resulting in residual Ni impurities [19, 20]. Furthermore, cylindrical lithium‐ion batteries commonly employ Ni‐plated steel cans for corrosion resistance [21]; when such cans are recycled, the Ni coating is readily dissolved together with the Fe substrate during leaching, further contributing to residual Ni in recycled Fe streams. As a result, recycled Fe‐based precursors frequently contain Fe and Ni in comparable chemical states.

From a chemical‐processing perspective, the selective separation of Fe and Ni species is particularly challenging due to their similar aqueous chemistry and overlapping redox and coordination behavior. Achieving efficient separation typically requires multi‐step oxidation control or solvent‐extraction schemes, significantly increasing process complexity and associated environmental burden [22, 23]. These practical constraints underscore the importance of understanding whether residual Ni can be tolerated within the LFP lattice, rather than assuming complete impurity removal as a prerequisite for high‐performance cathode synthesis.

The concept of “impurities as functional dopants” has been demonstrated in industrial FeSO_4_ derived from TiO_2_ production [24], where trace elements such as Ti, Mg, and Al were shown to suppress Li/Fe antisite defects and enhance electronic conductivity in LFP [25, 26, 27]. These studies suggest that impurity effects are highly element‐specific and concentration‐dependent rather than universally detrimental. Despite these insights, the roles of Ni and Cr—the primary impurity elements in STS‐derived and battery‐recycling‐derived Fe precursors—remain insufficiently understood. Although several studies have reported Ni‐doped LFP, mechanistic understanding of defect formation and electronic‐structure evolution remains limited, and such studies are rarely supported by density functional theory (DFT) calculations [28, 29]. Moreover, the structural and electrochemical consequences of Cr incorporation into the LFP lattice have not yet been systematically investigated.

Despite increasing interest in impurity‐containing precursors, a critical knowledge gap remains in understanding how realistic residual impurities—particularly Ni and Cr—interact with the LFP lattice under recycling‐relevant conditions. In particular, structural instability arising from lattice strain during electrochemical cycling has been widely recognized as a key factor governing cathode degradation [30]. Existing studies have largely focused on intentional doping strategies, often without systematically addressing thermodynamic feasibility, defect chemistry, and electronic‐structure evolution in an integrated manner. Moreover, the distinct roles of different impurity species have rarely been directly compared, and the behavior of Cr impurities in LFP remains especially underexplored. Here, we address this gap by combining comprehensive experimental characterization with DFT calculations to establish a mechanistic framework for impurity tolerance in LFP. By explicitly contrasting Ni and Cr, we reveal fundamentally different incorporation pathways—thermodynamically accessible substitution versus energetically unfavorable segregation—and their consequent impacts on lattice structure, electronic states, and lithium‐ion transport. Rather than pursuing idealized dopant design, this work defines realistic impurity‐tolerance limits under recycling‐derived precursor conditions, providing actionable guidelines for balancing purification requirements against electrochemical performance. These findings advance a new paradigm in which impurity management, rather than complete impurity elimination, enables cost‐effective and sustainable LFP synthesis from recycled resources.

## 2 Results and Discussion

### 2.1 Thermodynamic Feasibility of Transition‐Metal Incorporation in LFP

To assess the thermodynamic feasibility of transition‐metal incorporation in olivine‐type LiFePO_4_ (LFP), density functional theory (DFT) calculations were first performed. Because the incorporation of impurity elements during synthesis is governed by their energetic compatibility with the host lattice, the formation energy (E_F_) was employed as a quantitative descriptor to evaluate the likelihood of impurity incorporation [31, 32]. The formation energy was defined as:

$$E_{F}=E_{T,\;doped\;LFP}-\;E_{LFP}+n_{i}\left(\mu_{Fe}-\;\mu_{dopant}\right)$$

where *E*_*T*, *doped* *LFP*_ and *E_LFP_* are the total energies of the doped and pristine LFP supercells, respectively, *n_i_* is the number of substituted Fe atoms, and μ denotes the chemical potential of the corresponding elements. A lower formation energy indicates a more energetically accessible and structurally tolerable incorporation within the LFP lattice [33]. Here, the chemical potentials were defined with respect to the corresponding elemental bulk phases under Fe‐rich conditions, providing a consistent reference framework for comparing impurity incorporation energetics. While the formation energies are derived from total energy differences within a consistent reference framework, they primarily serve as comparative indicators of impurity incorporation tendencies rather than absolute thermodynamic quantities. In this study, the DFT analysis was restricted to substitution of dopants at the Fe site as a representative incorporation pathway. Possible occupation of Li sites by dopant species was not explicitly considered and remains to be investigated in future work.

Table 1 summarizes the calculated total energies (E_T_) and E_F_ of pristine LFP, Ni‐substituted LFP (LFP–Ni), and Cr‐substituted LFP (LFP–Cr) structures. While pristine LFP serves as the reference system, Ni substitution exhibits a relatively low formation energy of 0.103 eV, indicating that residual Ni species can be accommodated within the LFP lattice with only a minor energetic penalty [34]. Such a small E_F_ suggests that Ni incorporation is thermodynamically accessible under typical synthesis conditions and is unlikely to induce severe lattice destabilization [31, 35]. In contrast, Cr substitution yields a substantially higher formation energy of 1.214 eV, implying that Cr incorporation into the olivine framework is thermodynamically unfavorable and energetically costly. This large energetic penalty suggests that Cr incorporation is likely to be suppressed during synthesis and instead promotes phase segregation or structural instability [35].

**TABLE 1:** Total and formation energies of LFP, LFP–Ni, and LFP–Cr structure.

Structure	E_T_ [eV]	E_F_ [eV]
LiFePO_4_	−47786.489	—
LiNi_y_Fe_1‐y_PO_4_	−48274.722	0.103
LiCr_y_Fe_1‐y_PO_4_	−49389.655	1.214

These formation energies reflect thermodynamic tendencies and do not explicitly account for kinetic limitations during synthesis. These results indicate that, among common transition‐metal impurities encountered in recycled Fe‐based precursors, Ni is energetically far more likely to be accommodated within the olivine lattice than Cr. Accordingly, the subsequent sections primarily examine the structural, electronic, and electrochemical effects of residual Ni incorporation in LFP, with Cr serving as a comparative impurity case to elucidate impurity‐dependent lattice compatibility.

### 2.2 Structural, Electronic, and Electrochemical Effects of Residual Ni in LFP

**FIGURE 1:**
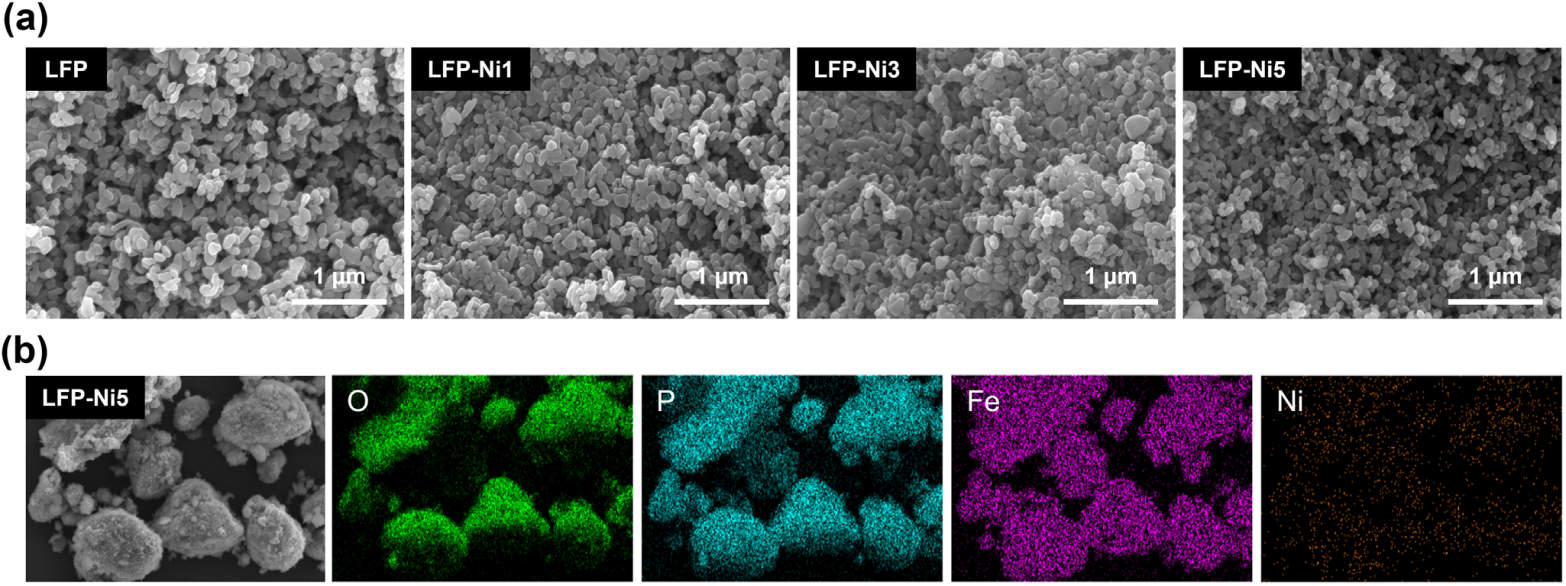
(a) SEM images of pristine LFP and Ni‐substituted LFP samples (LFP–Ni1, LFP–Ni3, and LFP–Ni5). (b) EDS elemental mapping images of LFP–Ni5 showing the spatial distribution of O, P, Fe, and Ni.

Guided by this prediction, the microstructural features of pristine LFP and Ni‐substituted LFP samples were examined by SEM–EDS to verify whether Ni incorporation indeed proceeds without inducing morphological variation or compositional inhomogeneity at the particle level. Hereafter, LFP–Nix (x = 1, 3, and 5) denotes LiFe_1−y_Ni_y_PO_4_, where y = 0.01, 0.03 and 0.05, corresponding to 1, 3, and 5 mol% substitution of Ni for Fe, respectively. As shown in Figure 1a, pristine LFP and all LFP–Ni samples exhibit a nanoplate‐like morphology with an average particle size of approximately 150 nm. No discernible differences in particle size or shape are observed across the investigated Ni substitution range up to 5%, indicating that Ni incorporation does not significantly alter particle growth behavior under the present synthesis conditions. EDS elemental mapping (Figure 1b) further shows that O, P, Fe, and Ni signals spatially overlap within individual LFP particles. No Ni‐rich agglomerates or segregated secondary phases are detected, even at the highest substitution level (LFP–Ni5), confirming homogeneous Ni distribution within the spatial resolution of EDS.

Following the computational prediction that Ni can be thermodynamically incorporated into the LFP lattice (Table 1) and the experimental confirmation of homogeneous Ni distribution at the particle level (Figure 1), the structural consequences of Ni substitution were investigated in detail by transmission electron microscopy (TEM) and X‐ray diffraction (XRD).

**FIGURE 2:**
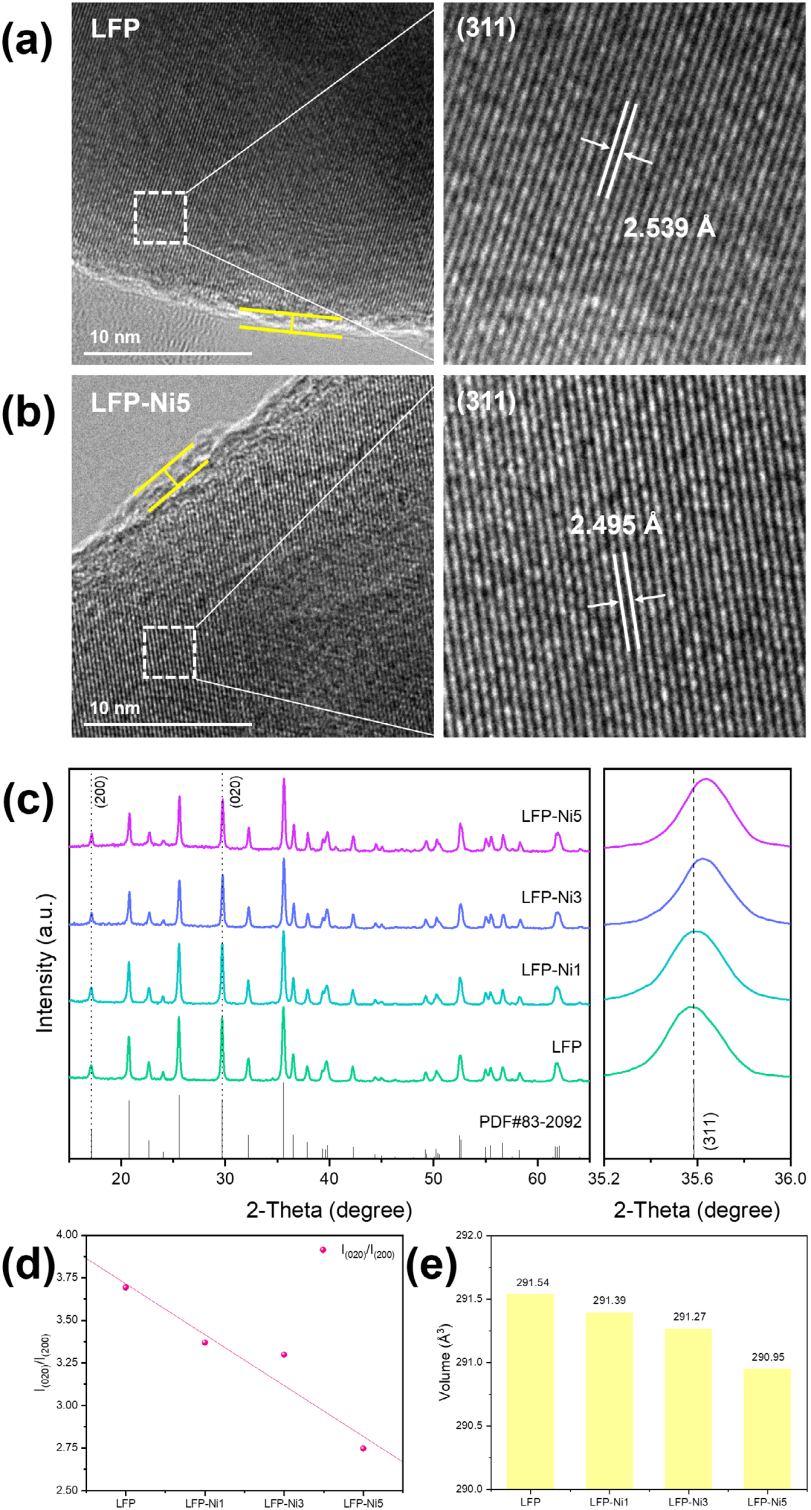
FE‐TEM images of (a) pristine LFP and (b) LFP–Ni5. (c) XRD diffraction patterns of pristine LFP and LFP–Ni samples, together with an enlarged view of the (311) peak region. (d) Diffraction intensity ratio of I_(020)_/I_(200)_ as a function of Ni content with a linear regression line. (e) Unit‐cell volume of LFP–Ni samples obtained from Rietveld refinement.

Figure 2a,b show FE‐TEM images of pristine LFP and LFP–Ni5, respectively. Because LFP intrinsically suffers from low electronic conductivity, carbon coating is commonly employed in practical LFP cathodes [36]. Both samples are uniformly coated with a thin amorphous carbon layer with a thickness of approximately 2 nm, indicating that Ni substitution does not affect carbon‐coating formation under the present synthesis conditions. No noticeable difference in carbon‐layer morphology is observed upon Ni substitution, indicating that Ni does not significantly influence carbon deposition behavior under the present synthesis conditions. High‐resolution TEM lattice‐fringe analysis reveals a clear contraction of the crystal lattice upon Ni substitution. The interplanar spacings were determined from the HRTEM images using fast Fourier transform (FFT) analysis, followed by lattice fringe measurement, and the reported values were averaged over multiple regions. Specifically, the interplanar spacing of the (311) plane decreases from 2.539 Å for pristine LFP to 2.495 Å for LFP–Ni5, indicating lattice contraction. This behavior is consistent with partial substitution of Fe^2+^ ions (0.78 Å) by smaller Ni^2+^ ions (0.69 Å) [37, 38].

The bulk crystallographic evolution was further examined by XRD (Figure 2c). All diffraction peaks of pristine LFP and LFP–Ni samples are well indexed to the olivine‐type LFP structure with an orthorhombic *Pnma* space group (JCPDS No. 83–2092), and no secondary phases such as NiO or Ni_2_P are detected [37]. With increasing Ni content, the diffraction peaks exhibit a gradual shift toward higher 2θ angles, as highlighted in the enlarged (311) reflection, confirming progressive lattice contraction at the bulk scale [37, 38]. Importantly, no additional diffraction peaks or peak broadening associated with crystalline or amorphous Ni‐containing secondary phases are observed within the detection limit of XRD.

Given that lithium‐ion transport in olivine‐type LFP occurs along the one‐dimensional [010] direction, crystallographic characteristics associated with this direction were evaluated using the diffraction intensity ratio I_(020)_/I_(200)_ (Figure 2d) [39, 40]. Because the (020) plane is perpendicular to the [010] Li^+^ diffusion channel, the I_(020)_/I_(200)_ ratio serves as an indicator of preferential orientation related to the exposure and alignment of diffusion pathways. The ratio decreases monotonically from 3.694 for pristine LFP to 3.370, 3.299, and 2.748 for LFP–Ni1, LFP–Ni3, and LFP–Ni5, respectively, indicating a gradual weakening of preferential orientation along the [010] direction with increasing Ni content [41].

To quantitatively substantiate these observations, Rietveld refinement was performed (Figure S1), and the refined structural parameters are summarized in Table S1 and visualized in Figure 2e. The unit‐cell volume decreases systematically from 291.540 Å^3^ for pristine LFP to 291.391, 291.265, and 290.949 Å^3^ for LFP–Ni1, LFP–Ni3, and LFP–Ni5, respectively, providing quantitative confirmation of lattice contraction induced by Ni substitution. The low refinement residuals (R_p_ = 1.30–1.46, R_wp_ = 1.73–1.87) indicate good agreement between the experimental data and the olivine LFP structural model. Taken together, the combined evidence from EDS, TEM, and XRD provides consistent multiscale support for the substitutional incorporation of Ni into the LFP lattice. The absence of Ni‐rich domains in EDS, the lattice contraction observed at the atomic scale in TEM, and the systematic peak shift and unit‐cell shrinkage in XRD collectively rule out surface segregation or amorphous phase formation within the detection limits of the employed techniques. These observations strongly indicate that Ni is incorporated at the Fe sites within the olivine framework.

**FIGURE 3:**
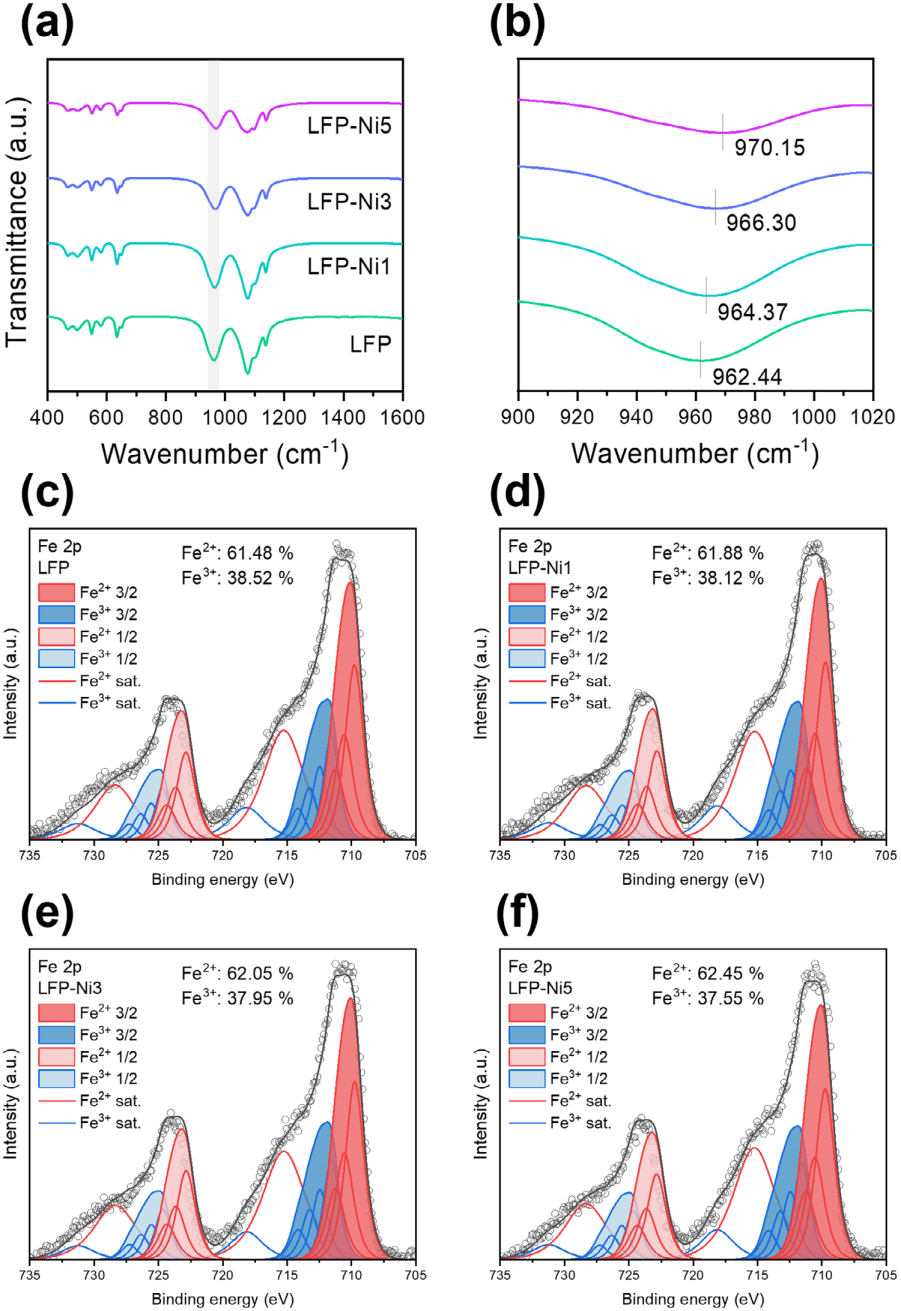
(a) FT‐IR spectra of pristine LFP and LFP–Ni samples. (b) Enlarged view of the P–O symmetric stretching vibration region. (c–f) Fe 2p XPS spectra of pristine LFP, LFP–Ni1, LFP–Ni3, and LFP–Ni5.

To probe changes in the bonding environment induced by Ni substitution, Fourier‐transform infrared (FT‐IR) spectroscopy was performed (Figure 3a). All samples exhibit the characteristic FT‐IR features of olivine‐type LFP, indicating that the fundamental phosphate framework is preserved upon Ni incorporation. Among these features, the absorption band in the 945–1139 cm^−1^ region is assigned to the symmetric stretching vibration of P–O bonds in PO_4_^3−^ tetrahedra and is known to be sensitive to lattice distortion and defect chemistry in LFP [42]. In particular, the position of this vibration band has been widely used as a spectroscopic indicator of Li/Fe antisite defects and structural perturbations [27, 43, 44].

As shown in Figure 3b, the P–O symmetric stretching band exhibits a systematic shift toward higher wavenumbers with increasing Ni content, appearing at 962.44, 964.37, 966.30, and 970.15 cm^−1^ for pristine LFP, LFP–Ni1, LFP–Ni3, and LFP–Ni5, respectively. In defect‐free LFP, this band is typically located near 957 cm^−1^. The progressive blue shift therefore indicates an increasing degree of structural distortion associated with antisite‐related defects upon Ni substitution [27, 43, 44]. This trend is consistent with the lattice contraction identified by XRD (Figure 2), indicating that cumulative unit‐cell shrinkage leads to subtle modifications of the Fe/Ni–O–P framework through changes in bond lengths and bond angles. Such structural distortions can influence the geometry of the Li‐ion diffusion channels, and have been widely reported to impede lithium‐ion transport along the one‐dimensional [010] pathways in olivine‐type LiFePO_4_ [45, 46]. A plausible origin of the increased Li/Fe antisite defects at higher Ni contents is the cumulative lattice contraction induced by substitution of larger Fe^2+^ ions with smaller Ni^2+^ ions. This contraction likely introduces local strain and perturbs the cation coordination environment, which can alter the energetic balance between Li and Fe site occupancy and thereby promote antisite defect formation during synthesis, consistent with previous studies showing that local structural distortion and cation‐site perturbation can influence antisite defect formation in transition‐metal oxides [30, 47].

To investigate the effect of Ni substitution on the Fe electronic states in LFP, X‐ray photoelectron spectroscopy (XPS) was performed. Figure 3c–f show the high‐resolution Fe 2p spectra of pristine LFP and LFP–Ni samples. The Fe 2p spectra consist of Fe 2p_3/2_ and Fe 2p_1/2_ components arising from spin–orbit splitting, with a fixed separation of 13.1 eV.

Quantitative analysis was carried out using the Fe 2p_3/2_ region. The spectra were deconvoluted using a constrained multiplet fitting scheme, in which the Fe^2+^ contribution was represented by three multiplet components and the Fe^3+^ contribution by four multiplet components. When the respective multiplet components are summed, the resulting Fe^2+^ and Fe^3+^ main envelopes are centered at approximately 710 and 712 eV, respectively, consistent with reported Fe^2+^/Fe^3+^ binding energies for olivine‐type LFP [48, 49]. Shake‐up satellite features were included to reproduce the overall spectral shape but were excluded from quantitative analysis.

The extracted Fe^2+^ fractions for pristine LFP, LFP–Ni1, LFP–Ni3, and LFP–Ni5 are 61.48%, 61.88%, 62.05%, and 62.45%, respectively. A slight, composition‐dependent increase in the fitted Fe^2+^ fraction is observed with increasing Ni content, suggesting a corresponding decrease in the relative Fe^3+^ contribution. Given that Fe^3+^ species in LFP are commonly associated with surface oxidation and non‐equilibrium surface states, these results are consistent with a tendency for Ni incorporation to mitigate surface oxidation and stabilize the Fe^2+^‐dominant surface environment. Notably, while XRD and FT‐IR indicate that excessive Ni substitution promotes bulk lattice distortion and antisite‐related defects (Figures 2 and 3a,b), the XPS analysis points to a potentially favorable surface‐related effect of Ni substitution—namely, a reduced contribution from surface Fe^3+^ species [50].

To provide a microscopic electronic‐structure‐based interpretation of the surface Fe electronic‐state evolution observed by XPS (Figure 3c–f), DFT calculations were performed. The calculations focus on how Ni substitution modifies local metal–oxygen bonding and the associated electronic charge redistribution within the LFP lattice.

**FIGURE 4:**
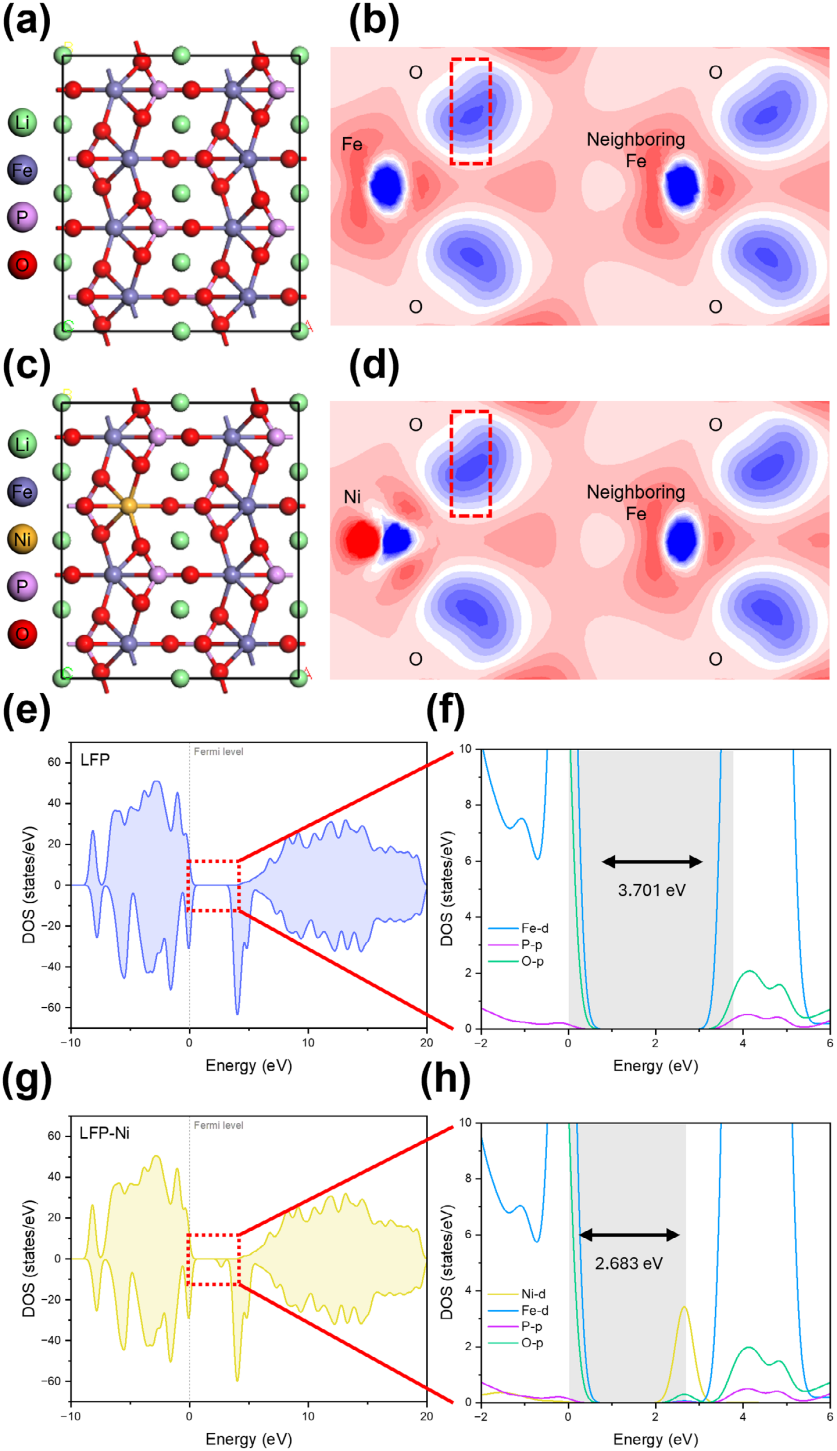
Atomic framework structures, corresponding charge density difference maps for (a,b) pristine LFP and (c,d) LFP–Ni. Blue and red regions in the charge density difference maps represent electron accumulation and electron depletion, respectively. Total density of states (DOS) and partial density of states (PDOS) near the Fermi level for (e,f) pristine LFP and (g,h) LFP–Ni.

Figure 4a,c presents the atomic framework structures of pristine LFP and the Ni‐substituted LFP supercell, respectively, in which a single Fe atom is replaced by Ni. Because Fe and Ni do not form direct metal–metal bonds in the olivine lattice, any electronic influence of Ni substitution on neighboring Fe sites must be mediated through the shared oxygen‐coordinated network [51]. To visualize such effects, charge density difference maps were constructed on bc‐plane slices positioned at half the distance between selected transition‐metal atoms and their coordinating oxygen atoms. In these maps, blue and red regions represent electron accumulation and electron depletion, respectively.

The corresponding charge density difference maps are shown in Figure 4b,d. In pristine LFP (Figure 4b), electron accumulation is primarily localized at oxygen centers, reflecting the strong electron‐attracting nature of oxygen within the Fe–O bonding framework. Upon Ni substitution (Figure 4d), electron accumulation at the oxygen atoms is further enhanced, as evidenced by the expanded blue regions at the oxygen centers.

Notably, an increase in electron accumulation is also observed at oxygen atoms bonded to neighboring Fe sites within the same crystallographic plane, indicating that the electronic influence of Ni substitution extends beyond the immediate Ni–O coordination environment. This behavior indicates that Ni substitution promotes a redistribution of electronic density toward oxygen‐centered regions through the shared oxygen sublattice, accompanied by a modification of the local electronic environment around neighboring Fe sites.

Such a redistribution, characterized by a shift of bonding electron density toward the oxygen sites, is expected to reduce electronic screening around the Fe ions [52]. As a consequence, the Fe‐centered electrons that participate in oxidation are stabilized by a stronger local electrostatic potential, which can make their removal during oxidation energetically less favorable [53]. In this context, the charge density difference analysis provides a microscopic electronic picture that is consistent with the reduced fraction of surface Fe^3+^ species observed in XPS measurements.

The electronic structure was further examined through total and partial density‐of‐states (DOS and PDOS) analyses (Figure 4e–h). The total DOS of pristine LFP and LFP–Ni remains largely unchanged, indicating preservation of the overall bulk electronic framework upon Ni incorporation. However, the PDOS reveals the emergence of Ni‐derived electronic states near the band‐gap region, located close to the Fe 3d band edge. Importantly, the calculated band gap decreases from 3.701 eV for pristine LFP to 2.683 eV for Ni‐substituted LFP, indicating that Ni‐derived states introduce intermediate energy levels within the band gap. Such states near the band edge can contribute to enhanced charge‐transfer kinetics by lowering the electronic transport barrier, particularly at low Ni concentrations. However, depending on their energetic position and local concentration, these states may also act as localized trap states that hinder electronic conduction [31].

**FIGURE 5:**
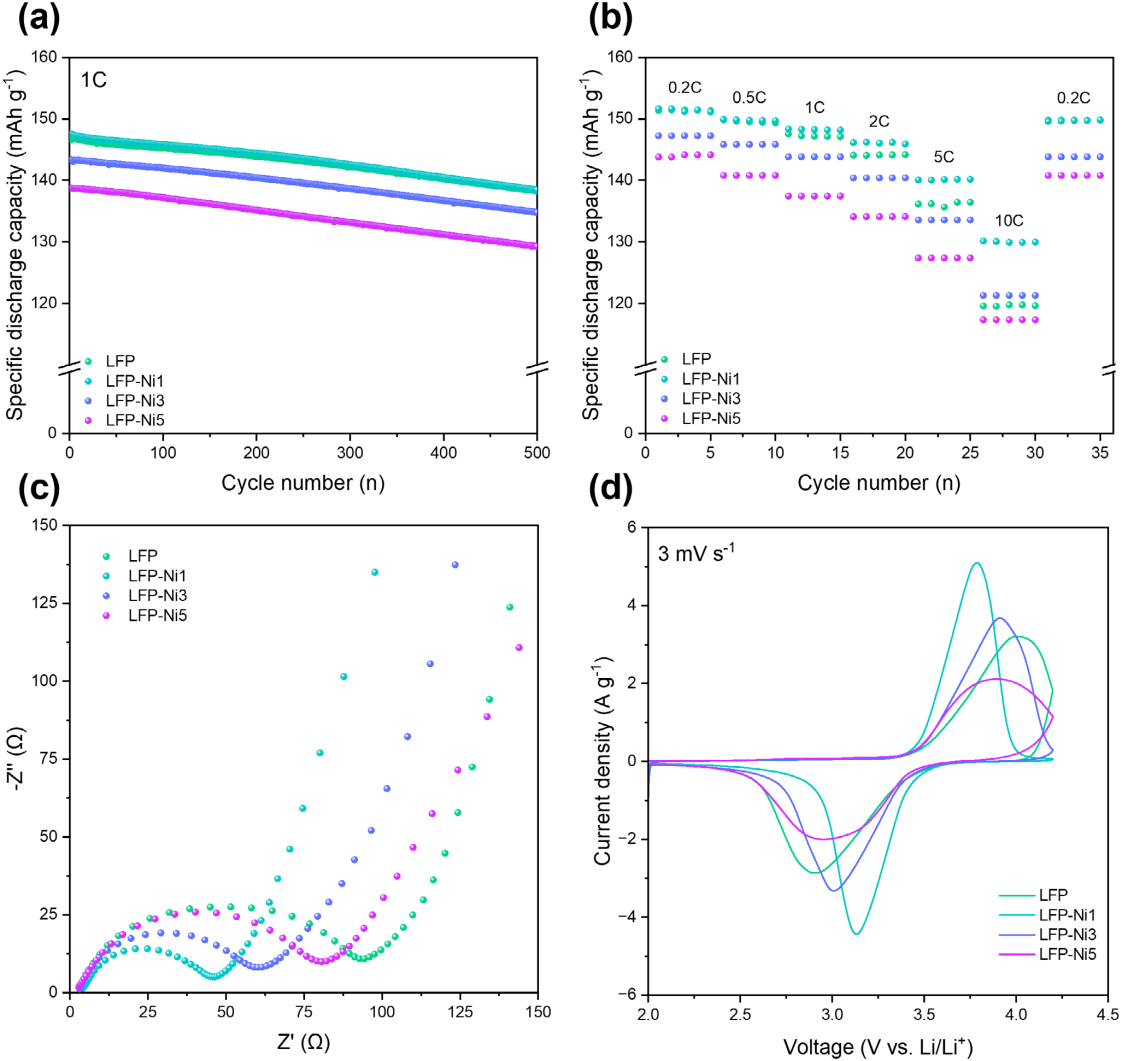
Electrochemical performance of pristine LFP and Ni‐substituted LFP samples (LFP, LFP–Ni1, LFP–Ni3, and LFP–Ni5). (a) Galvanostatic charge–discharge cycling performance at 1 C. (b) Rate performance measured at various C‐rates (0.2, 0.5, 1, 2, 5, and 10 C). (c) Electrochemical impedance spectroscopy (EIS) Nyquist plots recorded in the frequency range from 10^6^ to 0.01 Hz. (d) Cyclic voltammetry (CV) curves measured between 2.0 and 4.2 V at a scan rate of 3 mV s^−1^.

Figure 5a shows the galvanostatic charge–discharge cycling performance of pristine LFP and LFP–Ni electrodes measured at 1 C (1 C = 170 mAh g^−1^) within a voltage window of 2.5–4.2 V over 500 cycles. Among the investigated samples, LFP–Ni1 delivers the highest discharge capacity of 147.8 mAh g^−1^. Because the Ni^2+^/Ni^3+^ redox couple lies outside the operating voltage range of LFP [54], Ni does not contribute directly to capacity. Accordingly, the discharge capacity gradually decreases with increasing Ni content due to the replacement of electrochemically active Fe sites. Even when the discharge capacity is normalized by Fe content to exclude the contribution of electrochemically inactive Ni (Figure S2), LFP–Ni5 still exhibits a lower effective capacity than pristine LFP, indicating that excessive Ni substitution does not provide intrinsic capacity benefits.

Figure 5b compares the rate performance of pristine LFP and LFP–Ni electrodes. LFP–Ni1 exhibits the best high‐rate capability, delivering 130 mAh g^−1^ at 10 C. In contrast, LFP–Ni5 shows a pronounced capacity decay, indicating that excessive Ni substitution adversely affects reaction kinetics. Notably, the extended dataset (Figure S3) reveals that the rate capability begins to decrease at LFP–Ni2, with further deterioration observed at higher Ni contents, indicating that kinetic degradation starts beyond 1 mol% Ni.

Electrochemical impedance spectroscopy (EIS) measurements were conducted to further examine kinetic differences among the samples (Figure 5c). The Nyquist plots show that LFP–Ni1 exhibits the smallest semicircle in the high‐to‐medium frequency region, corresponding to the lowest charge‐transfer resistance (R_ct_), whereas pristine LFP and LFP–Ni5 display larger semicircles, indicating slower interfacial charge‐transfer processes.

To quantitatively assess lithium‐ion transport, the lithium‐ion diffusion coefficient (D_Li+_) was estimated from the low‐frequency Warburg region of the EIS spectra, where solid‐state diffusion dominates the impedance response [55]. To this end, the Warburg coefficient (σ) was obtained from the linear fitting of the real part of the impedance (Z_re_) as a function of ω^−1/2^ (Figure S4), according to:

$$Z_{re}=R_{S}+R_{ct}+\sigma \omega^{-1/2}$$

Using the extracted σ values, the lithium‐ion diffusion coefficient was calculated using the following relation [25]:

$$D_{Li^{+}}=\frac{R^{2}T^{2}}{2A^{2}n^{4}F^{4}C^{2}\sigma^{2}}$$

where R is the gas constant, T is the absolute temperature (298.15 K), A is the electrode surface area, n is the number of electrons involved in the Fe^2+^/Fe^3+^ redox reaction (n = 1), F is Faraday's constant, and C is the molar concentration of lithium ions in LFP. The extracted values of R_ct_, σ, and D_Li+_ are summarized in Table 2. LFP–Ni1 exhibits a higher diffusion coefficient (2.73 × 10^−14^ cm^2^ s^−1^) than pristine LFP (1.58 × 10^−14^ cm^2^ s^−1^), confirming enhanced lithium‐ion transport at low Ni substitution levels. The extended dataset (Figure S3) further shows that both R_ct_ and D_Li+_ progressively deteriorate from LFP–Ni2 onward, indicating that the transition from beneficial to detrimental effects occurs between 1 and 2 mol% Ni.

**TABLE 2:** Electrochemical impedance spectroscopy (EIS)–derived parameters, including charge‐transfer resistance (R_ct_), Warburg coefficient (σ), and lithium‐ion diffusion coefficient (D_Li+_), for pristine LFP and Ni‐substituted LFP samples.

Sample	Mass Loading [mg cm^−2^]	R_ct_ [Ω]	σ [Ω·s^−1/2^]	D_Li+_ [cm^2^·s^−1^]
LFP	1.57	95.89	42.12	1.58 × 10^−14^
LFP‐Ni1	1.79	47.06	30.54	2.73 × 10^−14^
LFP‐Ni3	1.91	57.56	47.84	1.24 × 10^−14^
LFP‐Ni5	1.97	80.65	60.86	7.61 × 10^−15^

Cyclic voltammetry (CV) measurements were further employed as an independent electrochemical probe to assess redox kinetics and polarization behavior (Figure 5d). At a scan rate of 3 mV s^−1^, LFP–Ni1 exhibits sharper and more symmetric redox peaks with a smaller peak separation, indicating improved redox reversibility and reduced polarization. In contrast, LFP–Ni5 shows broadened peaks and increased peak separation, consistent with sluggish charge‐transfer kinetics.

To further evaluate the impact of antisite defects at the cell level, memory‐effect tests were conducted for pristine LFP, LFP–Ni1, and LFP–Ni5 (Figure S5). In this test, a memory‐writing step was first applied by interrupting the charge–discharge process at a specific state of charge, followed by a memory‐releasing cycle in which the voltage profile was compared with that of a normal full cycle. The magnitude of the memory effect is evaluated by examining whether the memory‐releasing voltage profile deviates from the normal cycle, particularly through the appearance of additional voltage steps, plateau distortion, or enhanced hysteresis [56].

Li–Fe antisite defects are known to disrupt the synchronicity of the two‐phase transition between LiFePO_4_ and FePO_4_, leading to non‐uniform lithiation and delithiation during partial cycling [44, 57, 58]. Because lithium transport in olivine‐type LFP proceeds along one‐dimensional [010] channels, even a small degree of antisite disorder can induce diffusion heterogeneity and phase‐transition asynchrony, which manifests as a voltage‐memory effect during subsequent cycling.

Consistent with this mechanism, pristine LFP exhibits the weakest memory effect: the memory‐releasing voltage profile nearly overlaps with the normal cycle, indicating minimal retention of the written state and a highly uniform phase transition. LFP–Ni1 shows a slightly increased but still limited memory effect, characterized by a subtle distortion of the voltage plateau, suggesting that moderate Ni substitution introduces only minor diffusion heterogeneity. In contrast, LFP–Ni5 displays pronounced voltage hysteresis and severely distorted plateaus during the memory‐releasing cycle, with clear deviations from the normal profile. These features provide direct electrochemical evidence of substantial antisite‐induced diffusion non‐uniformity and phase‐transition asynchrony.

Overall, the electrochemical behavior observed in Figure 5 can be consistently interpreted by integrating the structural, spectroscopic, and computational results presented in Figures 1, 2, 3, 4. Pristine LFP serves as a structurally stable reference with minimal antisite disorder and uniform lithium‐ion diffusion. At low Ni substitution levels, surface Fe oxidation is suppressed and charge‐transfer kinetics are enhanced through Ni‐derived electronic states, while bulk structural distortion and antisite defects remain limited. This balance results in optimal electrochemical performance for LFP–Ni1.

A quantitative comparison of kinetic and transport parameters further clarifies this balance. At low Ni substitution levels, both charge‐transfer resistance and lithium‐ion diffusion behavior indicate enhanced interfacial kinetics and ion transport. However, with increasing Ni content, this trend reverses, with progressively hindered lithium‐ion transport arising from bulk structural distortion and antisite defects.

These results demonstrate that the electrochemical performance is governed by a trade‐off between surface kinetic enhancement and bulk transport degradation. While low‐level Ni substitution (∼1 mol%) maximizes this balance, the extended dataset (Figure S3) reveals that the transition from beneficial to detrimental behavior occurs between 1 and 2 mol% Ni, beyond which bulk structural distortion and antisite defects increasingly dominate the electrochemical response.

Previous studies have reported that transition‐metal dissolution from cathodes followed by migration and deposition on the anode can occur during prolonged cycling, potentially contributing to additional interfacial impedance growth [59]. In this context, such cross‐electrode effects may also be relevant in the present system.

### 2.3 Structural Incompatibility and Electrochemical Degradation Induced by Cr Impurities

Having demonstrated that residual Ni species originating from stainless‐steel‐derived precursors can be accommodated within the LFP lattice without severe structural or electrochemical degradation, we next examine the behavior of Cr, another common impurity element, to evaluate impurity tolerance and phase stability during LFP synthesis from non‐ideal Fe sources.

**FIGURE 6:**
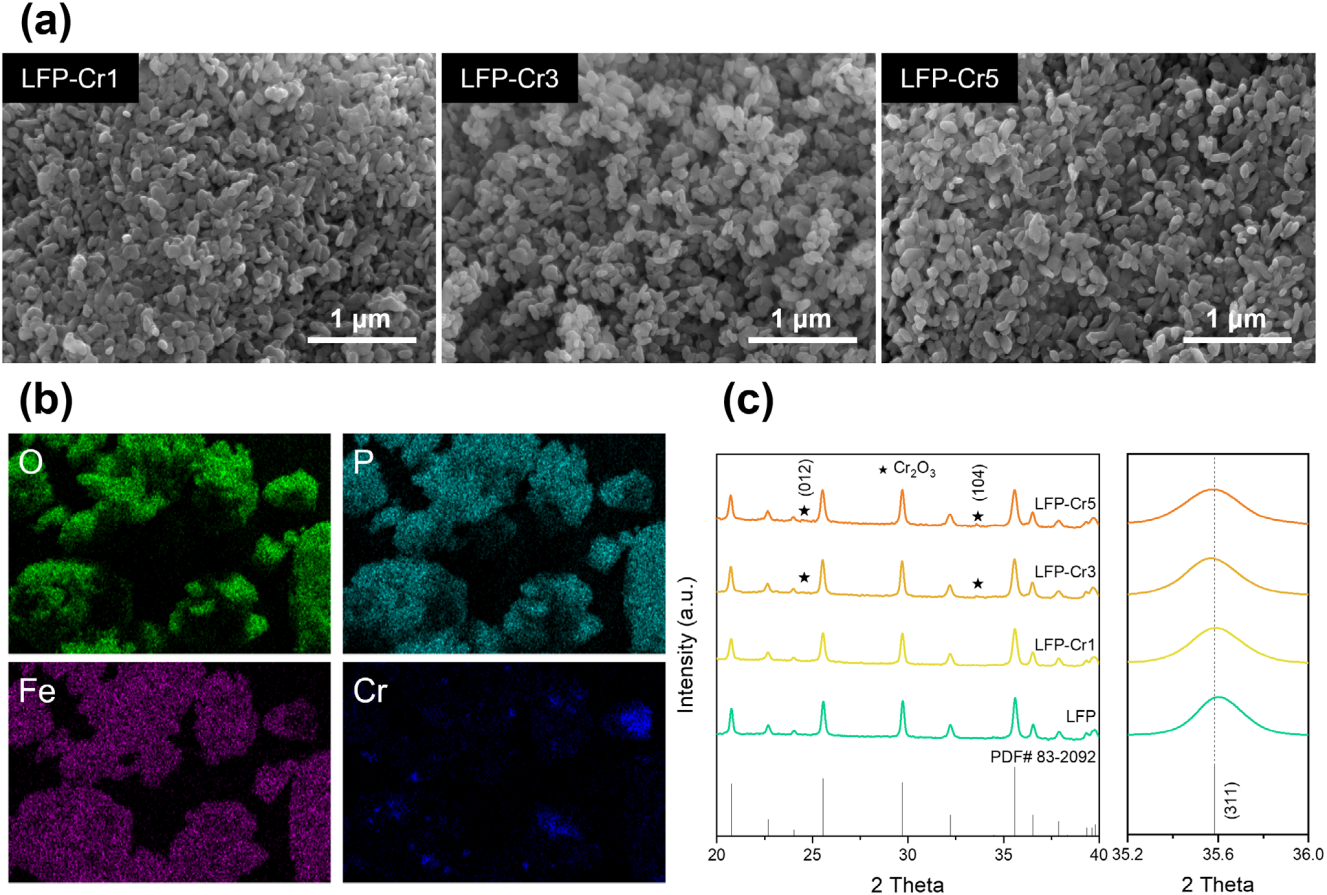
(a) SEM images of LFP, LFP–Cr1, LFP–Cr3, and LFP–Cr5, (b) EDS mapping of LFP–Cr5 (O, P, Fe, and Cr), (c) XRD diffraction peaks of all LFP–Cr samples, together with an enlarged view of the (311) peak region.

To examine whether residual Cr species induce morphological changes in LFP particles, SEM analysis was performed (Figure 6a). All LFP–Cr samples exhibit a nanoplate‐like morphology similar to that of pristine LFP, with no discernible differences in particle shape or size. This observation indicates that the presence of Cr impurities does not significantly alter particle growth behavior under the present synthesis conditions.

To assess the incorporation behavior of Cr within the LFP particles, elemental distribution was further analyzed by EDS mapping (Figure 6b). In contrast to the homogeneous distribution observed for residual Ni species, Cr exhibits a clearly inhomogeneous spatial distribution, characterized by localized Cr‐rich regions with significantly higher Cr signal intensity compared to the surrounding matrix. This aggregation behavior suggests that Cr is poorly accommodated within the LFP lattice and instead tends to segregate as Cr‐containing secondary phases. Such inhomogeneous distribution is fully consistent with the formation‐energy calculations (Table 1), which predict that Cr incorporation into the LFP lattice is thermodynamically unfavorable, and is further corroborated by the XRD and Rietveld results discussed below.

The structural consequences of Cr impurities were further evaluated by XRD analysis (Figure 6c). While the primary diffraction peaks of all LFP–Cr samples can still be indexed to the olivine‐type LFP structure, weak additional reflections corresponding to Cr_2_O_3_ ((012) and (104)) are detected [60], indicating the formation of Cr‐containing secondary phases outside the LFP lattice [61]. In addition, the relative intensity of Cr_2_O_3_ peaks increases with increasing Cr content, further supporting progressive phase segregation. Notably, the characteristic (311) reflection of olivine‐type LFP appears at 35.60°, 35.58°, 35.56°, and 35.58° for LFP, LFP–Cr1, LFP–Cr3, and LFP–Cr5, respectively. Although minor variations are observed, no systematic peak shift is detected with increasing Cr content, and the changes remain within the typical instrumental resolution (∼0.02°). This absence of a consistent peak shift indicates that Cr does not induce measurable lattice distortion and is therefore not incorporated into the LFP lattice. To further quantify this structural deviation, Rietveld refinement was performed using a single‐phase olivine LFP model (Figure S6). R_wp_ increases from 1.79 for pristine LFP to 2.24, 2.72, and 2.83 for LFP–Cr1, LFP–Cr3, and LFP–Cr5, respectively, indicating progressively poorer agreement with the LFP structural model as the Cr content increases. Although the weak Cr_2_O_3_ reflections are insufficient for reliable phase‐fraction quantification, this systematic increase in R_wp_ provides quantitative support for the structural incompatibility of Cr with the LFP lattice. Taken together, these structural analyses consistently demonstrate that Cr is not incorporated into the LFP lattice but instead segregates as Cr_2_O_3_ secondary phases, leading to increasing structural deviation from the ideal olivine framework.

**FIGURE 7:**
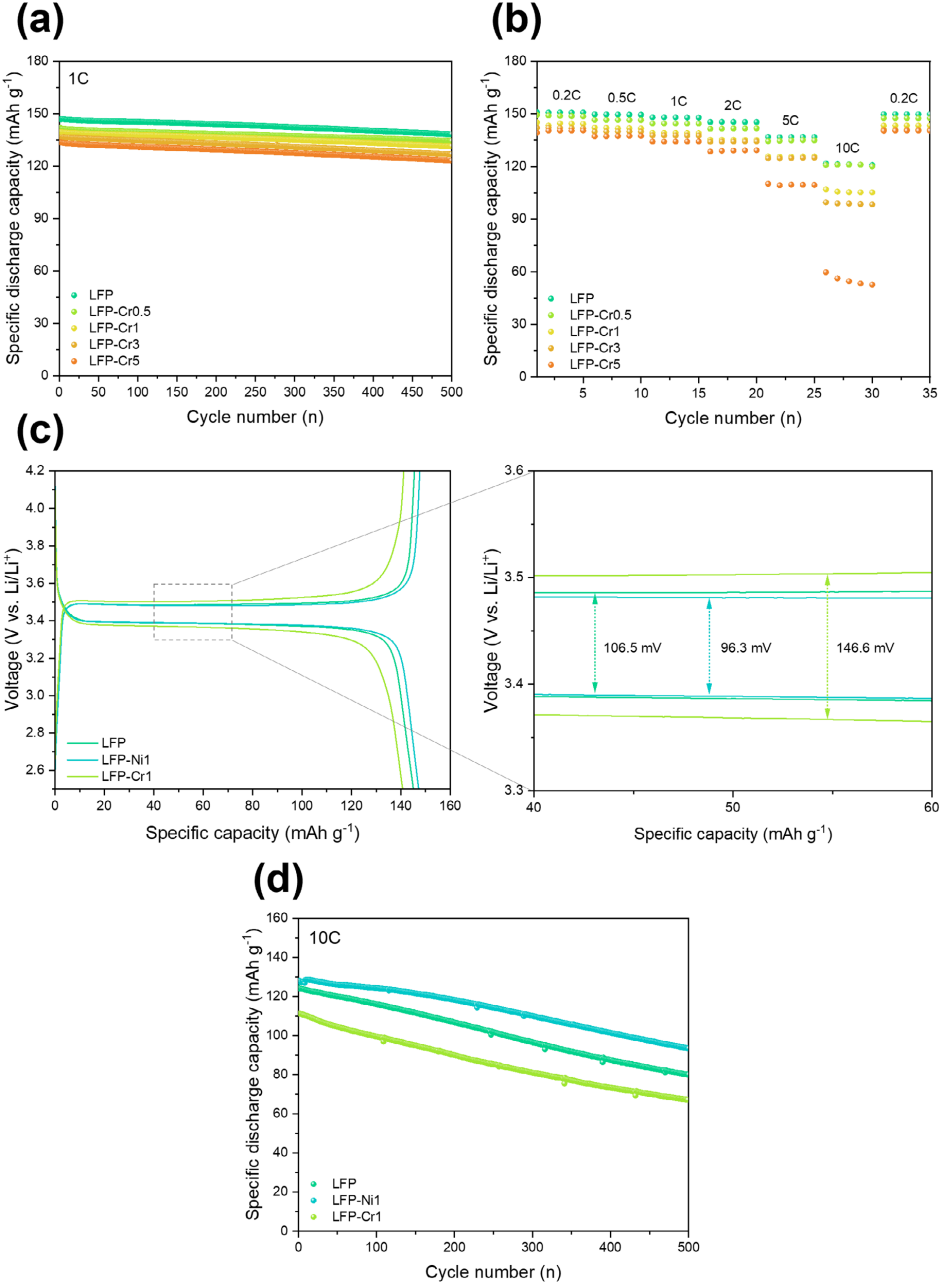
Electrochemical performance of pristine LFP and LFP–Cr samples. (a) Galvanostatic charge–discharge cycling performance measured at 1 C. (b) Rate performance measured at various C‐rates (0.2, 0.5, 1, 2, 5, and 10 C). (c) Galvanostatic charge–discharge voltage profiles at 1 C comparing pristine LFP, LFP–Ni1, and LFP–Cr1, with enlarged views highlighting voltage hysteresis and polarization behavior. (d) Galvanostatic charge–discharge cycling performance measured at 10C comparing pristine LFP, LFP–Ni1, and LFP–Cr1.

The electrochemical impact of residual Cr species in LFP was evaluated through galvanostatic cycling and rate‐performance measurements (Figure 7a,b). At a current rate of 1 C, LFP–Cr1 delivers a discharge capacity of 139 mAh g^−1^, which is lower than that of pristine LFP and markedly inferior to the performance achieved by optimally Ni‐containing LFP. This reduced capacity reflects the limited electrochemical contribution of Cr‐related species and is consistent with the poor lattice compatibility inferred from formation‐energy calculations and structural analyses, which prevents lattice incorporation and promotes the formation of electrochemically inactive Cr_2_O_3_ secondary phases.

The rate‐performance results shown in Figure 7b further highlight the detrimental effect of Cr impurities on kinetic behavior. While pristine LFP maintains moderate capacity retention with increasing C‐rate, Cr‐containing samples exhibit a pronounced deterioration in high‐rate performance. In particular, LFP–Cr5 shows a severe capacity drop, delivering only 60 mAh g^−1^ at 10 C, indicating that increasing Cr content exacerbates kinetic limitations rather than facilitating lithium‐ion transport. This behavior is consistent with the presence of segregated Cr_2_O_3_ secondary phases identified by EDS and XRD analyses. The localized Cr‐rich regions observed by EDS suggest that these Cr_2_O_3_ phases exist as discrete aggregates and may partially cover the surface of LFP particles, thereby potentially reducing the effective electrode–electrolyte contact area. As an electrochemically inactive phase with low ionic and electronic conductivity, Cr_2_O_3_ does not contribute to lithium storage and instead acts as a resistive barrier within the electrode, leading to diffusion‐blocking effects and degraded rate capability. Such partial obstruction of active LFP surfaces can further disrupt lithium‐ion transport pathways and increase interfacial resistance.

To gain further insight into reaction kinetics, polarization behavior was analyzed using galvanostatic charge–discharge voltage profiles at 1 C (Figure 7c), together with enlarged views highlighting the plateau regions. The overpotentials of pristine LFP, LFP–Ni1, and LFP–Cr1 were determined to be 106.5, 96.3, and 146.6 mV, respectively. The substantially higher overpotential observed for LFP–Cr1 indicates increased polarization and sluggish charge‐transfer kinetics, which can be attributed to disrupted lithium‐ion transport pathways and increased internal resistance caused by segregated Cr_2_O_3_ phases with intrinsically low ionic and electronic conductivity.

The detrimental influence of Cr impurities is further evidenced by high‐rate long‐term cycling at 10 C (Figure 7d). LFP–Ni1 exhibits superior cycling stability, delivering an initial discharge capacity of 128.17 mAh g^−1^ and retaining 93.53 mAh g^−1^ after 500 cycles. Pristine LFP shows more pronounced capacity fading, decreasing from 124.19 to 80.07 mAh g^−1^ over the same cycling period. In contrast, LFP–Cr1 displays the poorest cycling stability, with capacity fading from 111.32 mAh g^−1^ in the first cycle to only 67.27 mAh g^−1^ after 500 cycles. This accelerated degradation under sustained high‐rate cycling demonstrates that Cr impurities introduce persistent kinetic barriers that cannot be mitigated through repeated cycling.

Taken together, these results indicate that the detrimental effects of Cr impurities originate primarily from their intrinsic chemical incompatibility with the LFP lattice, which prevents lattice incorporation and drives the formation of Cr_2_O_3_ secondary phases. As an electrochemically inactive phase with low ionic and electronic conductivity, Cr_2_O_3_ acts as a resistive barrier, introducing microstructural diffusion‐blocking effects that lead to increased polarization and hindered lithium‐ion transport. Consequently, Cr impurities result in severe degradation of electrochemical performance.

## 3 Conclusions

In summary, this study systematically elucidates the contrasting roles of Ni and Cr impurities in LFP synthesized from non‐ideal Fe precursors by combining comprehensive experimental characterization with DFT calculations. The results demonstrate that impurity tolerance in LFP is not universal but strongly dependent on both elemental identity and concentration. Residual Ni species commonly encountered in recycled Fe‐based precursors can be accommodated within the LFP lattice at low concentrations without severe structural disruption. In particular, incorporation of 1 mol% Ni induces favorable modulation of the local electronic environment, including localized electronic polarization and the emergence of Ni‐related states near the Fe 3d band edge. These electronic effects, together with minimal lattice distortion and limited Li–Fe antisite defect formation, enhance charge‐transfer kinetics and lithium‐ion diffusion, resulting in optimized discharge capacity and superior high‐rate performance. In contrast, Ni contents above the optimal level of ∼1 mol%, with the transition to detrimental behavior occurring between 1 and 2 mol%, lead to cumulative lattice contraction and an increase in antisite defects, which obstruct the one‐dimensional lithium‐ion diffusion channels intrinsic to the olivine structure and ultimately degrade electrochemical performance. Cr impurities exhibit fundamentally different behavior. Formation‐energy calculations reveal that Cr incorporation into the LFP lattice is thermodynamically unfavorable, and experimental analyses confirm inhomogeneous Cr distribution and segregation of electrochemically inactive Cr_2_O_3_ secondary phases. The lack of lattice incorporation, combined with pronounced phase segregation, introduces resistive and diffusion‐blocking heterogeneities that result in increased polarization, hindered lithium‐ion transport, and markedly inferior electrochemical performance compared with both pristine and Ni‐containing LFP. Collectively, these findings demonstrate that exhaustive and energy‐intensive purification of recycled precursors is not always necessary to achieve high‐performance LFP cathodes. Instead, a rational impurity‐tolerance strategy—based on quantitative assessment of impurity species, concentration, and lattice compatibility—can provide practical guidelines for the chemical valorization of industrial byproducts and recycled battery materials. This work therefore offers a viable pathway toward cost‐effective and environmentally responsible cathode‐material design for sustainable energy‐storage applications.

## Author Contributions

**Minjin Kim**: Conceptualization, Investigation, Methodology, Writing – original draft. **Hyerin Jeon**: Investigation, Validation. **Jinhee Lee**: Investigation, Methodology, Validation. Writing – review and editing. **Jinsub Choi**: Conceptualization, Writing – review and editing, Supervision, Funding acquisition.

## Conflicts of Interest

The authors declare no conflicts of interest.

## Supporting information




**Supporting File**: advs75774‐sup‐0001‐SuppMat.docx.

## Data Availability

The data that support the findings of this study are available from the corresponding author upon reasonable request.
