# Supplementary material for: Impurity Tolerance in LiFePO4 Cathodes: Contrasting Structural, Electronic, and Electrochemical Roles of Residual Ni and Cr
Source: Adv Sci (Weinh). 2026 May 22:e75774. Online ahead of print. doi: 10.1002/advs.75774 (PMC13336001; doi:10.1002/advs.75774)
Supplement: Supplementary file 1 — Supporting File: advs75774‐sup‐0001‐SuppMat.docx. [file ADVS-9999-e75774-s001.docx]

**Supporting information**

**Impurity Tolerance in LiFePO_4_ Cathodes: Contrasting Structural, Electronic, and Electrochemical Roles of Residual Ni and Cr**

*Minjin Kim ^a^, Hyerin Jeon^a^, Jinhee Lee^b, *^, Jinsub Choi^a, b, *^*

*^a^* Department of Chemistry and Chemical Engineering, Inha University, Incheon, 22212, Republic of Korea

*^b^* Department of Secondary Battery Convergence Engineering, Inha University, Incheon, 22212, Republic of Korea

^*^To whom correspondence should be addressed

Tel.: +82−32−860−7476

E-mail: jinhee333@inha.ac.kr; jinsub@inha.ac.kr

**Experimental**

**Material preparation**

Pristine LiFePO_4_ (LFP) and Ni-substituted LiFe_1−x_Ni_x_PO_4_ (LFP–Ni) samples were synthesized via a solvothermal method. First, 0.072 mol of LiOH·H_2_O was dissolved in ethylene glycol, followed by the addition of 0.036 mol of H_3_PO_4_ under continuous magnetic stirring. After the formation of a white suspension, 0.0048 mol of ascorbic acid was introduced to suppress the oxidation of Fe^2+^. Subsequently, a total of 0.024 mol of FeSO_4_·7H_2_O and NiSO_4_·6H_2_O, with Ni molar fractions ranging from 0 to 0.05 (corresponding to pristine LFP to LFP–Ni5), was added, and the mixture was stirred for 1 h.

The resulting dark blue–green solution was transferred into a 100 mL Teflon-lined stainless-steel autoclave and maintained at 180 °C for 6 h with a heating rate of 5 °C min^−1^. After natural cooling to room temperature, the precipitate was collected, washed several times with ethanol and deionized water by centrifugation, and dried at 80 °C for 12 h. For carbon coating, the dried powders were thoroughly ground with 20 wt% citric acid and subsequently annealed at 700 °C for 6 h under an Ar atmosphere, using a heating rate of 5 °C min^−1^.

**Materials characterization**

The morphology and elemental distribution of the synthesized LFP samples were examined using field-emission scanning electron microscopy (FE-SEM, S-4300, Hitachi) equipped with energy-dispersive X-ray spectroscopy (EDS, EX-250, Horiba). Detailed microstructural features were further investigated by field-emission transmission electron microscopy (FE-TEM, JEM-2100F, JEOL). Crystal structure and phase purity were analyzed by X-ray diffraction (XRD, SmartLab SE, Rigaku), and Rietveld refinement was performed using the Materials Studio software package. The local bonding environments were characterized using Fourier-transform infrared spectroscopy (FT-IR, VERTEX 80V, Bruker). Surface chemical states and valence information were examined by X-ray photoelectron spectroscopy (XPS, K-Alpha, Thermo Scientific).

**Coin cell fabrication and Electrochemical measurements**

For electrochemical measurements, the active material (LFP or LFP–Ni), Super C (conductive carbon), and polyvinylidene fluoride (PVDF) binder were mixed in a mass ratio of 8:1:1 using N-methyl-2-pyrrolidone (NMP) as the solvent. The resulting slurry was uniformly cast onto aluminum foil and dried at 120 °C for 3 h under vacuum. The electrodes were then pressed and further dried for an additional 6 h. The active material loading was controlled within the range of 1.6–2.0 mg cm^−2^.

Circular electrodes with a diameter of 14 mm were punched and assembled into CR2032-type coin cells in an argon-filled glovebox. Lithium metal foil was used as the counter electrode, with 1 M LiPF_6_ dissolved in a mixed solvent of ethylene carbonate and diethyl carbonate (EC/DEC, 1:1 v/v) as the electrolyte, and a Celgard 2400 polypropylene membrane as the separator. Prior to electrochemical testing, all cells were aged at 25 °C for 48 h and precycled for three cycles at 0.2 C within a voltage window of 2.5–4.2 V (vs. Li/Li^+^). Galvanostatic cycling and rate-performance tests were carried out using a battery cycler (CT-4008T, SinoproMRX). Cyclic voltammetry (CV) measurements were performed between 2.0 and 4.2 V using a Wonatech WBCS3000S1 system. Electrochemical impedance spectroscopy (EIS) was conducted over a frequency range of 10^5^ to 0.01 Hz using a potentiostat/galvanostat (Autolab PGSTAT128N, Metrohm).

**Computational details**

First-principles calculations were performed using the CASTEP module implemented in the Materials Studio software package. The exchange–correlation interactions were treated within the generalized gradient approximation (GGA) using the Perdew–Burke–Ernzerhof (PBE) functional. Ultrasoft pseudopotentials were employed to describe the electron–ion interactions for all constituent elements [1].

To model Ni substitution in LiFePO4, a 2 × 2 × 1 orthorhombic supercell containing 112 atoms was constructed. This supercell size was selected to provide a balanced cell geometry and to minimize artificial directional bias in defect–defect interactions, thereby approximating the dilute defect limit [2]. The Ni-substituted structure, LiNi_1/16_Fe_15/16_PO_4_, corresponds to a Ni concentration of 6.25 mol% at the Fe sites. A plane-wave cutoff energy of 480 eV was used, and the Brillouin zone was sampled with a 3 × 4 × 3 Monkhorst–Pack k-point mesh.

Geometry optimizations were performed until the following convergence criteria were satisfied: (i) the maximum atomic force was below 0.03 eV Å^−1^, (ii) the total energy change per atom was less than 1 × 10^−5^ eV, and (iii) the maximum ionic displacement was below 0.003 Å. The chemical potentials of Fe, Ni, and Cr were referenced to their respective elemental bulk phases, corresponding to Fe-rich boundary conditions. All defect calculations were performed assuming charge-neutral substitution at the Fe sites. All calculations were carried out in reciprocal space to evaluate the formation energies, density of states (DOS), and charge-density distributions of pristine and Ni-substituted LFP systems.


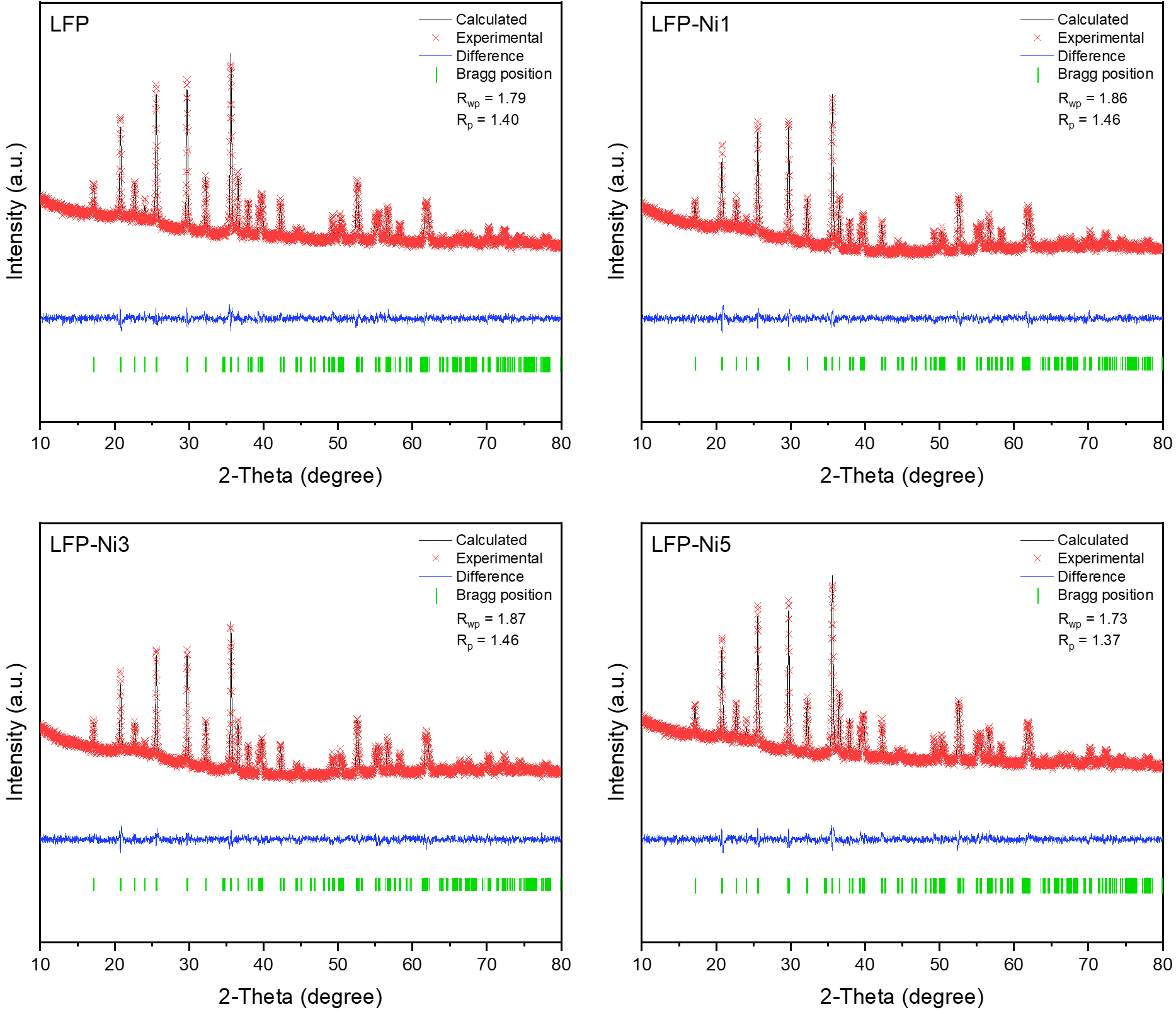


**Figure S1.** XRD Rietveld refinement profiles of pristine LFP and Ni-substituted LFP samples (LFP–Ni1, LFP–Ni3, and LFP–Ni5).

**Table S1.** Refined lattice parameters (a, b, c), unit-cell volume, and reliability factors obtained from Rietveld refinement for pristine LFP and LFP–Ni samples.

| Sample | a (Å) | b (Å) | c (Å) | Volume (Å^3^) | R_p_ | R_wp_ |
| --- | --- | --- | --- | --- | --- | --- |
| LFP | 10.33182 | 6.00875 | 4.69610 | 291.540 | 1.40 | 1.79 |
| LFP–Ni1 | 10.32983 | 6.00789 | 4.69527 | 291.391 | 1.46 | 1.86 |
| LFP–Ni3 | 10.32978 | 6.00684 | 4.69409 | 291.265 | 1.46 | 1.87 |
| LFP–Ni5 | 10.32664 | 6.00535 | 4.69158 | 290.949 | 1.37 | 1.73 |


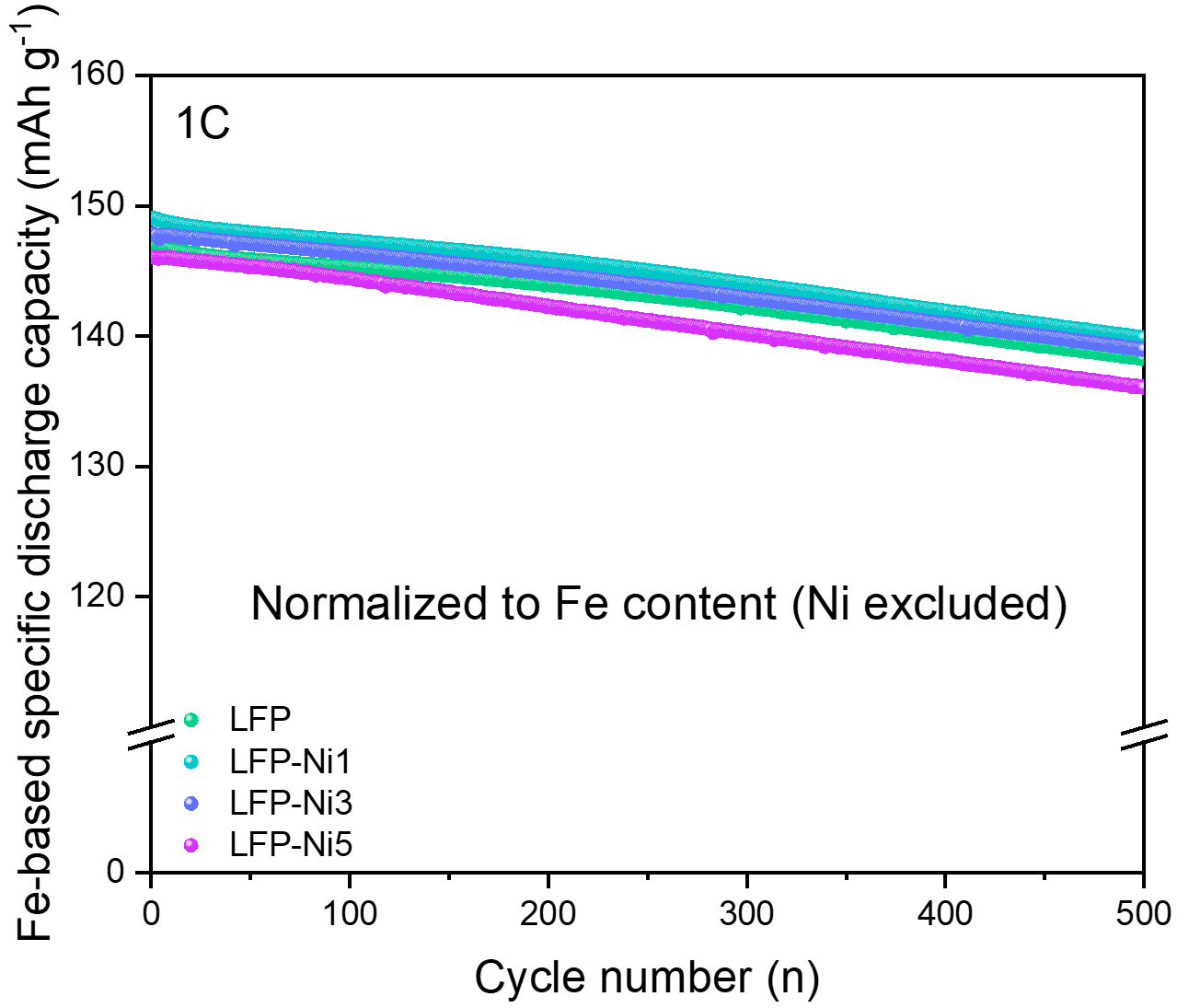


**Figure S2.** Galvanostatic charge–discharge cycling performance of pristine LFP and LFP–Ni samples at 1C. The specific capacity was normalized to the Fe content, excluding electrochemically inactive Ni.


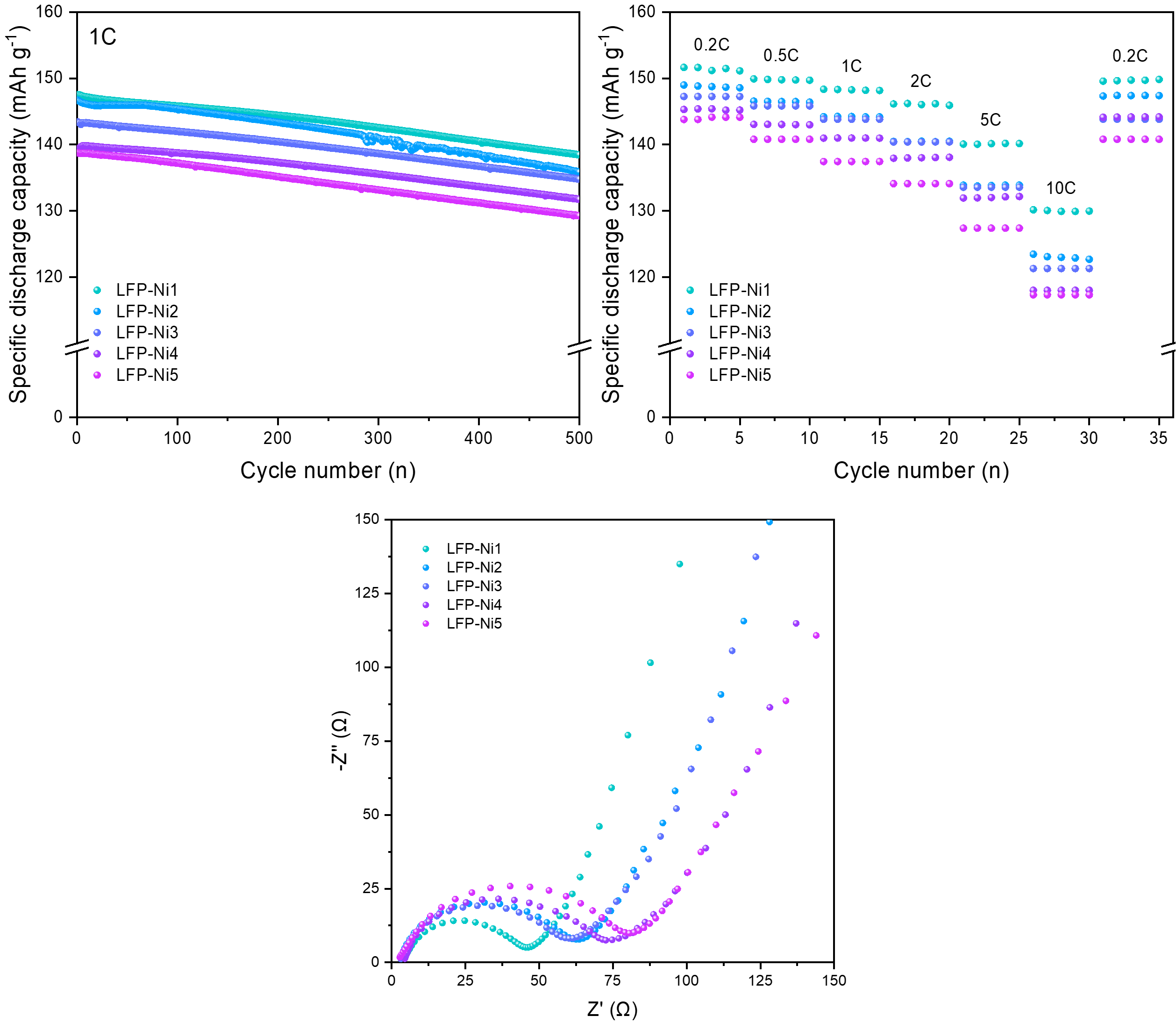


**Figure S3.** Extended electrochemical performance of Ni-substituted LFP samples (LFP–Ni1, LFP–Ni2, LFP–Ni3, LFP–Ni4, and LFP–Ni5). (a) Galvanostatic charge–discharge cycling performance at 1C. (b) Rate performance measured at various C-rates (0.2, 0.5, 1, 2, 5, and 10C). (c) Electrochemical impedance spectroscopy (EIS) Nyquist plots recorded in the frequency range from 10^6^ Hz to 0.01 Hz.


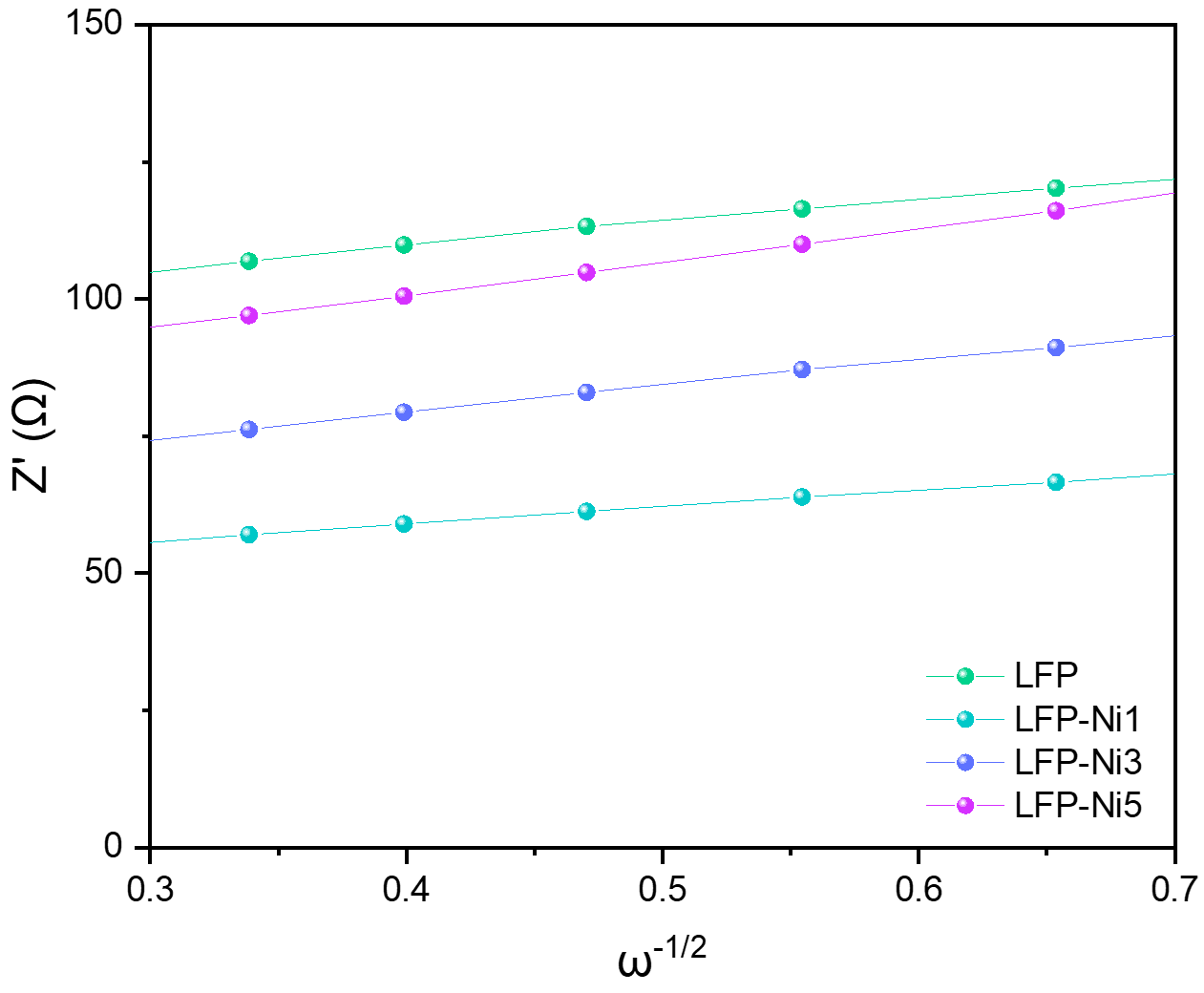


**Figure S4.** Linear fitting plots of the real part of impedance (Z′) versus ω^−1/2^ used to determine the Warburg coefficient (σ) for pristine LFP and LFP–Ni.


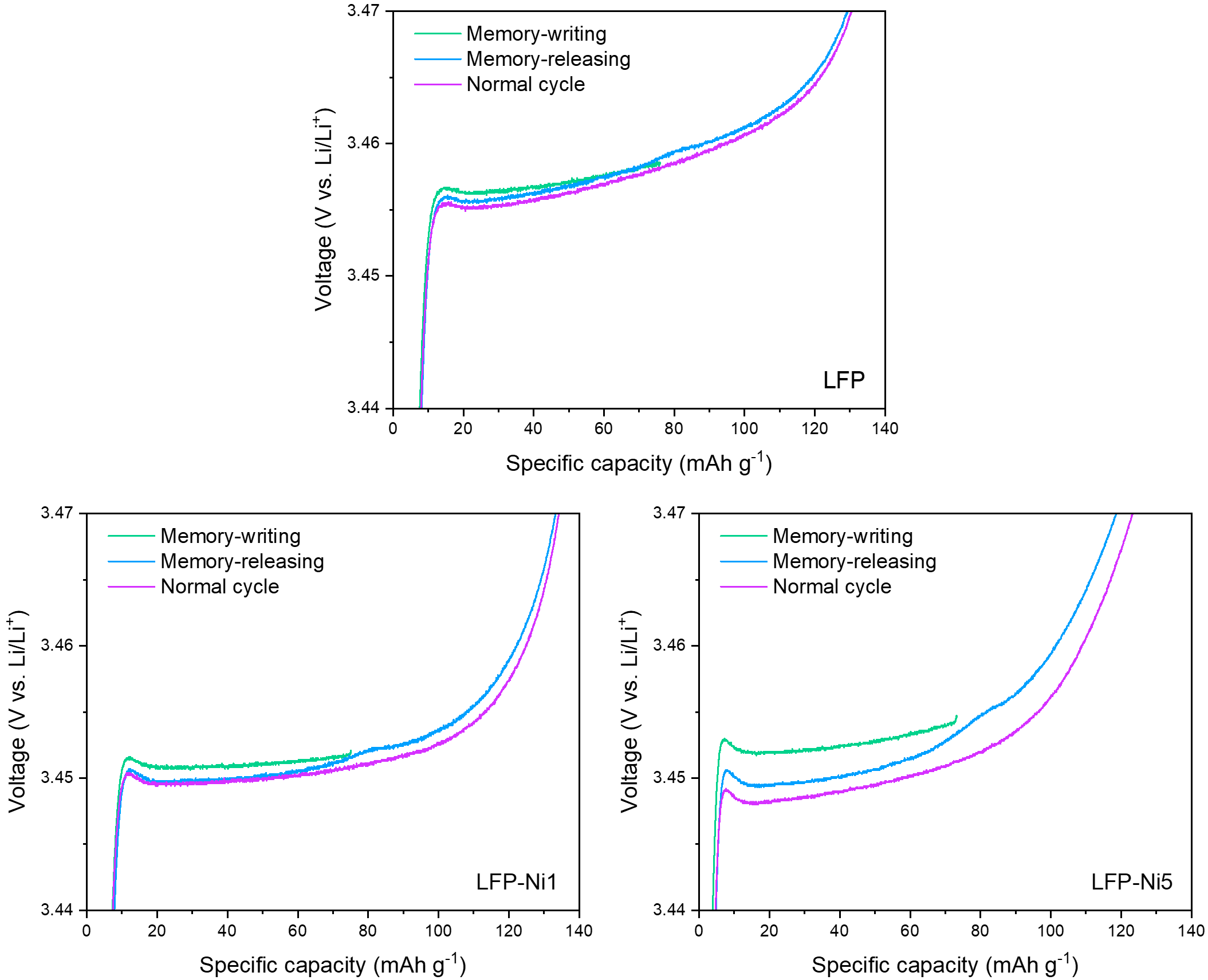


**Figure S5.** Memory-effect test results for pristine LFP, LFP–Ni1, and LFP–Ni5 electrodes. Half-charge curves during memory writing (green), full-charge curves during memory release (blue), and normal charge–discharge curves (purple) measured at 0.2C.


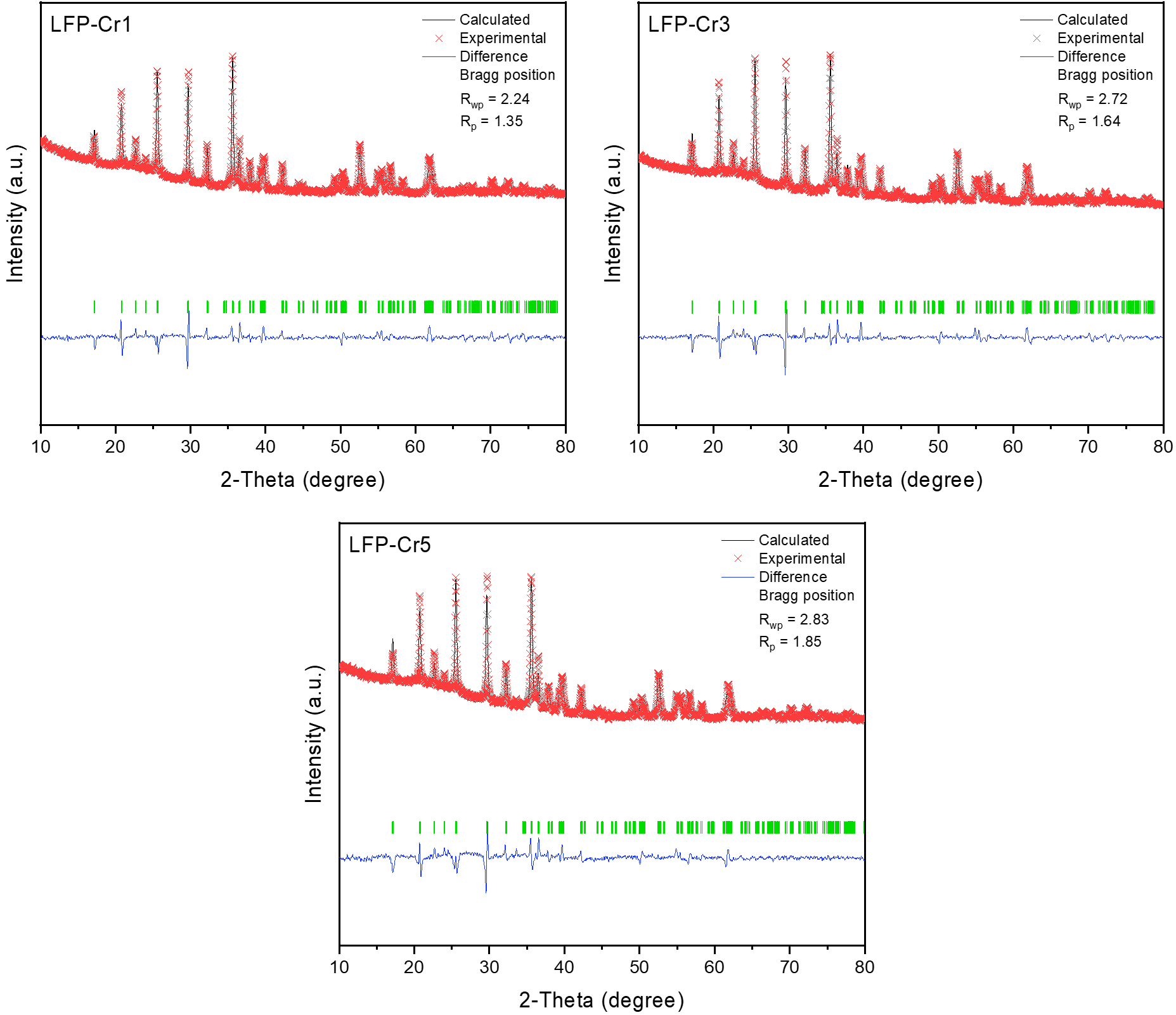


**Figure S6.** XRD Rietveld refinement profiles of Cr-substituted LFP samples (LFP–Cr1, LFP–Cr3, and LFP–Cr5).

**References**

[1] X. Zou, T. Liu, W. Liu, Y. Li, Y. Zhao, "First principles calculations on stability, electronic structure and fracture failure of Cu-doped Al(100)/Mg2Si(111) interface," *Mater. Chem. Phys.* (2024): 128978. <https://doi.org/10.1016/j.matchemphys.2024.128978>

[2] B. Lama, A. L. Smirnova, T. R. Paudel, "Enhanced Li-Ion Diffusivity of LiFePO4 by Ru Doping: Ab Initio and Machine Learning Force Field Results," *ACS Appl. Energy Mater.* (2023): 10424–10431. <https://doi.org/10.1021/acsaem.3c01429>
